# Anaphylactic shock following minor abdominal trauma as the initial presentation of Echinococcus cyst: a case report

**DOI:** 10.1186/s12887-022-03154-z

**Published:** 2022-02-12

**Authors:** Damla Hanalioglu, Kivanc Terzi, Sati Ozkan, Mesut Sivri, Funda Kurt, Emine Dibek Misirlioglu

**Affiliations:** 1 Department of Pediatrics, Division of Pediatric Emergency Medicine, University of Health Sciences, Ankara Child Health and Diseases, Hematology Oncology Training and Research Hospital, Ankara, Turkey; 2 Department of Pediatrics, Division of Pediatric Emergency Medicine, Hacettepe University Ihsan Dogramaci Children’s Hospital, 06100, Sihhiye, Ankara, Turkey; 3 Department of Radiology, University of Health Sciences, Ankara Child Health and Diseases, Hematology Oncology Training and Research Hospital, Ankara, Turkey; 4 Department of Pediatrics, Division of Pediatric Allergy, University of Health Sciences, Ankara Child Health and Diseases, Hematology Oncology Training and Research Hospital, Ankara, Turkey

**Keywords:** Anaphylaxis, Case report, Hydatid cyst, Liver, Trauma

## Abstract

**Background:**

Anaphylaxis is a potentially life-threatening condition caused by a variety of triggers. However, anaphylaxis following an abdominal trauma is an exceptionally rare condition and could be the first and only sign of hepatic hydatid cyst, especially when no obvious etiology is present. Here, we present such a rare case and discuss relevant diagnostic and management strategy in light of the literature.

**Case report:**

This case report refers to a 17 year-old previously healthy girl admitted in our pediatric emergency department (ED) for syncope after a minor blunt abdominal trauma. She was hypotensive on admission and shortly after she developed urticaria and angioedema. She was diagnosed with anaphylaxis and treated immediately. Possible etiologies including drug or food ingestion, insect bite, and previous allergy/anaphylaxis history were excluded. After stabilization abdominal imaging was performed, which revealed a ruptured large hepatic hydatid cyst in the vicinity of biliary tree. Albendazole treatment was started and surgical resection was performed after clinical stabilization, which confirmed the cyst rupture into the biliary ducts. Patient recovered without complications after surgery and was discharged uneventfully.

**Conclusion:**

This case report highlights that hydatid cyst rupture should be included in the differential diagnosis of anaphylaxis without obvious etiology, particularly in regions where hydatid disease is endemic. Ruptured hydatid cyst leading to anaphylaxis requires timely diagnosis, management and emergent intervention.

## Background

Anaphylaxis is a severe and potentially life-threatening allergic reaction [1]. It is estimated that approximately one to four patients per 1000 emergency department visits occur due to anaphylaxis [2]. The common triggers of anaphylaxis in children include food, insect stings and drugs [3]. However, anaphylaxis following an abdominal trauma is an exceptionally rare condition and could be the first and only sign of hepatic hydatid cyst, especially when no obvious etiology is present [4].

Hydatid disease also known as cystic echinococcosis is a zoonotic disease. Infection occurs by ingestion of parasite eggs in contaminated food, water or soil, or through direct contact with animal hosts. Hydatid disease is endemic in certain parts of the world including Turkey. Infections with Echinococcus granulosus lead to one or more hydatid cysts located most often in the liver and may remain asymptomatic for years until hydatid cysts cause clinical signs [5]. Most frequent clinical symptoms of hepatic hydatid cysts, mainly due to the mass effect of the enlarging cyst, are abdominal pain, nausea and vomiting. Other nonspecific symptoms include anorexia, weakness and weight loss [3]. Ultrasonography imaging is usually first choice for the diagnosis of human hydatid disease [5–7]. Specific antibodies detected by serological test, indirect hemagglutination and histopathological examination of cyst contents may support diagnosis [5, 6]. Secondary bacterial infections and cyst rupture are important complications of the disease [5, 6]. Complication frequency ranges from 5 to 40%. Rupture of a hydatid cyst of liver into the biliary tree occurs in 6-21% of patients [8–10]. Cyst may be ruptured spontaneously as a result of increased intracystic pressure or following a trauma. Anaphylaxis due to hydatid cyst rupture may lead to rapid clinical decline and require emergency medical and surgical interventions. In the literature, anaphylaxis following blunt trauma has been described in only a few case reports [4, 11–16].

Here, we report an unusual case in which anaphylaxis following mild abdominal trauma was the only presentation of hydatid disease in a 17-year-old female patient.

## Case presentation

Previously healthy 17-year-old female patient was admitted to the pediatric emergency department because of syncope and hypotension after walking into the classroom door at school when she had hit upper right side of her abdomen. Emergency medical services were noticed and she was transported to the ED via an ambulance. She seemed weak and confused. Her vital signs at admission were as follows: blood pressure 80/35 mmHg, heart rate 120 beats/min, temperature 38.5 °C. Her oxygen saturation on room air was 98%. Upon arrival, she demonstrated an urticarial rash mainly on her face and trunk together with angioedema of the eyelids and lips, and had severe abdominal pain, which occurred in approximately 15-20 min after the trauma. We treated her immediately with 0.5 mg intramuscular 1/1000 epinephrine for anaphylactic shock and initiated intravenous fluid resuscitation. Her response to the initial dose of epinephrine was poor, so that a repeated dosing was required after 15 min. Eventually, her blood pressure was maintained around 110/70 mmHg, approximately 10 min after the second epinephrine dose, with this treatment regime and her hemodynamic status was stabilized soon after. She also received intravenous prednisolone (50 mg) and diphenhydramine (50 mg) to prevent late reaction. Laboratory work-up revealed leukocytosis without eosinophilia and a high C-reactive protein level. Her bilirubin level was mildly elevated and she had hypoalbuminemia. The other laboratory tests were within normal limits (Table 1).

**Table 1:** Laboratory Results

Laboratory parameter	Result	Reference values
Hemoglobin (g/dL)	13.1	12 -15.5
White blood cell count (/mm^3^)	25,000	3500 – 10,500
Neutrophil count (/mm^3^)	20,000	2100 – 6100
Eosinophil count (/mm^3^)	200	0 - 500
Platelet count (/mm^3^)	194,000	150,000 – 450,000
C-reactive protein (mg/dL)	15.5	0 - 0.5
Alanin-amino transpherase (U/L)	11	0 - 35
Aspartate- amino transpherase (U/L)	20	0 - 35
Total bilirubin (mg/dL)	1.58	0.3 - 1.2
Conjugated bilirubin (mg/dL)	0.37	0 – 0.2
Albumin (g/dL)	2.9	3.2 – 4.5
Blood culture	Negative	Negative

**Fig. 1:**
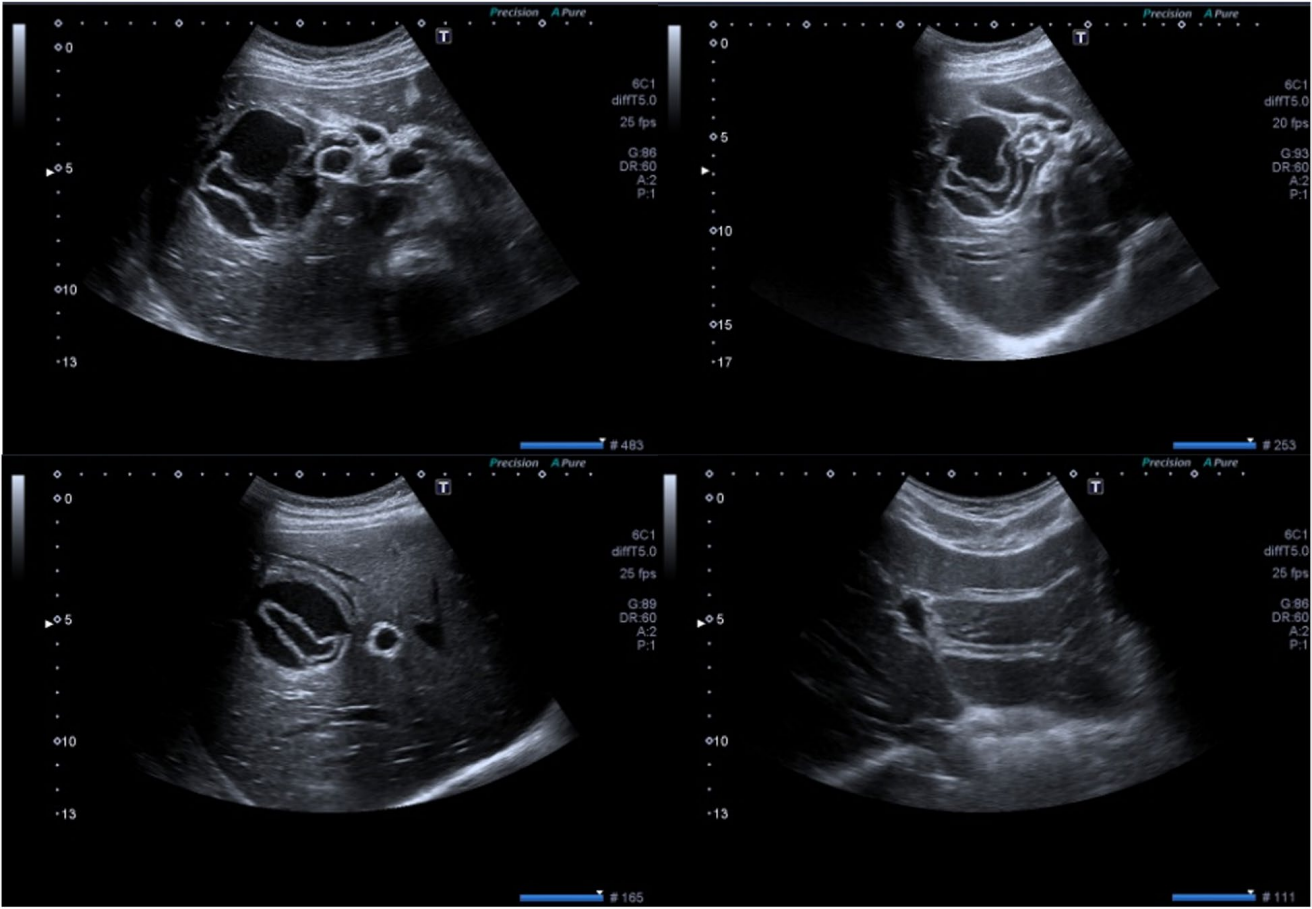
Abdominal ultrasonography images. Serial ultrasonographic images show type 2 ruptured hepatic hydatid cyst, inside of which floating membranes are present, located in segments five and six

**Fig. 2:**
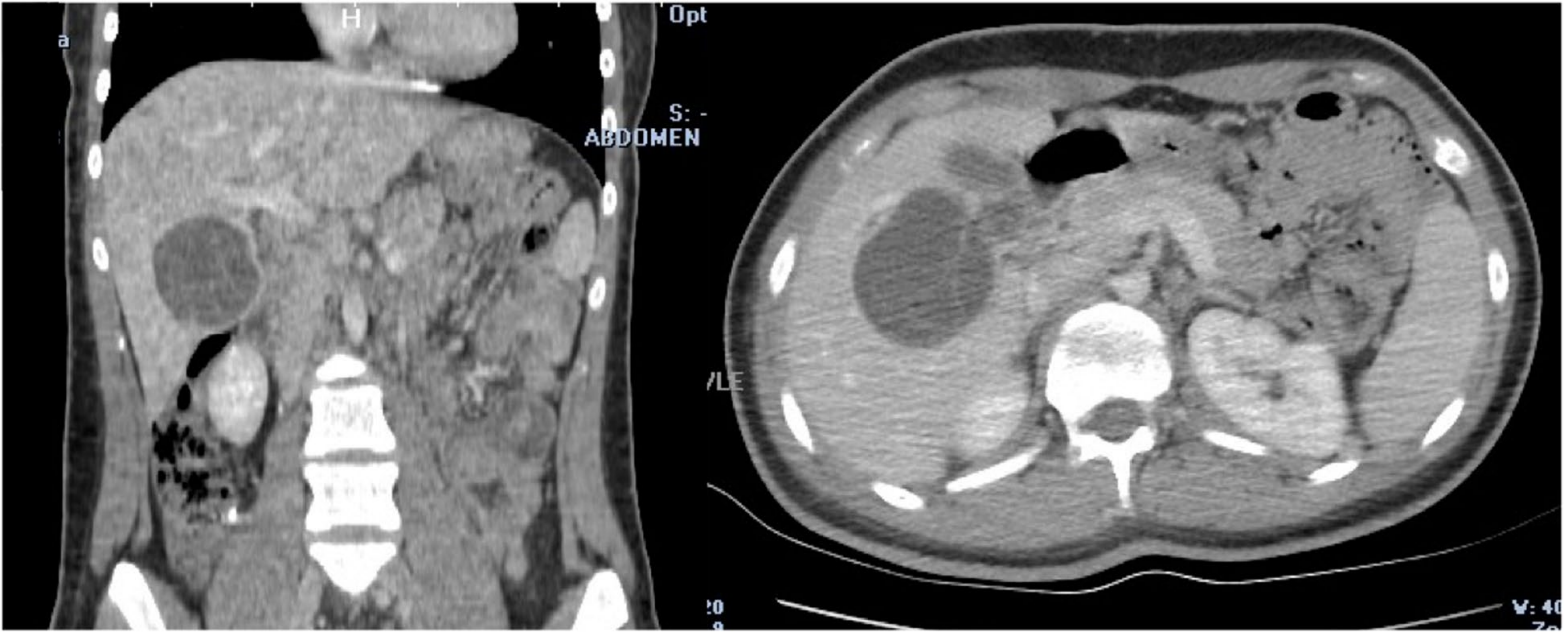
Abdominal computed tomography images. Image shows ruptured hepatic hydatid cyst, inside of which floating membranes are present

She and her parents both denied previous history of allergies, current medication use or food ingestion that could have clarified the etiology of a possible allergic reaction. There were no signs of insect bite marks. On suspicion that the anaphylactic shock was due to rupture of a hepatic hydatid cyst abdominal imaging was considered. Ruptured type 2 hydatid cyst (61 × 68 mm) located in the fifth and sixth segment of liver was evident on both abdominal ultrasonography (Fig. 1) and computed tomography (Fig. 2). Its close relation to biliary ducts raised suspicion of cyst rupture into the biliary tract. The patient was diagnosed with anaphylactic shock secondary to a ruptured liver hydatid cyst following minor blunt abdominal trauma. After administration of initial dose of anti-helmintic treatment with albendazole (10 mg/kg/day), the patient underwent emergent laparotomy the same day. Cyst rupture into the biliary tree was confirmed during surgery. Cyst membranes were excised, and ruptured cyst content were completely removed. Common bile duct was irrigated with 0.9% NaCl, and was surgically repaired. Surgical field was also irrigated with saline to evacuate any debris or cyst content. Hydatid cyst hemagglutination titer was 1/1280 (negative < 1/160). Histopathological examination showed hydatid cyst germinal membranes and scolices. The patient was discharged 1 week after surgery uneventfully. In order to reduce the risk of secondary hydatidosis, albendazole was continued for 6 months (10 mg/kg/day BID) after surgery. The patient recovered fully with this treatment and follow-up imaging at 3- and 6-months showed no signs of recurrence.

## Discussion and conclusions

We present an adolescent girl with previously unknown hydatid disease of the liver, which manifested with anaphylaxis due to cyst rupture following a minor blunt trauma to the abdomen. This patient was asymptomatic until this event. Most patients with hydatid disease remain silent for several years until cysts enlarge enough to trigger clinical symptoms mainly due to mass effect in a small space or until complications arise [5, 6]. Anaphylaxis, with or without cyst rupture, is a rare presentation to previously unknown cystic hydatid disease [4, 15, 16]. Therefore, high clinical suspicion is required for an accurate diagnosis and timely management in such cases like ours.

In our case, rupture and leakage to the biliary tree was evident in accordance with the previously reported cases. Like most authors argued, we think that antigen release following cyst rupture may trigger the anaphylactic reaction [4, 11–16]. Hydatid cysts located in the liver may rupture to peritoneal cavity or to biliary passages [9, 17]. Cyst-biliary communications were found to range between 6 and 21% of patients [8–10]. Early diagnosis and treatment of an intrabiliary rupture of a liver hydatid cyst is critical since mortality rate is 50% [9]. Our patient was successfully treated with adjuvant chemotherapy with albendazole, emergent surgical excision of the cyst and repair of bile ducts without any complications and recurrence. Most authors recommend a standard treatment that includes a combined treatment regime with anti-helmintic treatment and surgical resection or PAIR (Puncture, Aspiration, Injection, Re-aspiration) with satisfactory long-term clinical results [18].

This case report depicts a very rare case of minor blunt trauma-induced anaphylaxis caused by a rupture of a previously unknown hydatid cyst. We think that it is an informative and illustrative case for pediatricians, emergency and primary care physicians. This paper presents the diagnostic workup and clinical management process in detail. However, we were not able to obtain intraoperative picture or video, which could be demonstrative as well.

Hydatid cyst rupture should be considered in the differential diagnosis of anaphylaxis following blunt trauma as a missed or delayed diagnosis may lead to severe complications and sudden death.

## Data Availability

Not applicable.

## Abbreviations

ED: Emergency department
PAIR: Puncture, Aspiration, Injection, Re-aspiration

## References

[1] Dodd A, Hughes A, Sargant N, Whyte AF, Soar J, Turner PJ (2021). Evidence update for the treatment of anaphylaxis. Resuscitation.

[2] Alqurashi W, Stiell I, Chan K, Neto G, Alsadoon A, Wells G (2015). Epidemiology and clinical predictors of biphasic reactions in children with anaphylaxis. Ann Allergy Asthma Immunol.

[3] Shaker MS, Wallace DV, Golden DBK, Oppenheimer J, Bernstein JA, Campbell RL (2020). Anaphylaxis—a 2020 practice parameter update, systematic review, and grading of recommendations, assessment, development and evaluation (GRADE) analysis. J Allergy Clin Immunol.

[4] De Wispelaere L, Vande Velde S, Schelstraete P, Van Renterghem K, Moerman F, Van Biervliet S (2011). Anaphylactic shock as a single presentation of Echinococcus cyst. Acta Gastroenterol Belg.

[5] Kliegman. Echinococcosis (Echinococcus granulosus and Echinococcus Multilocularis). In: Nelson Textbook of Pediatrics. 20th edition.

[6] World Health Organization, Fact Sheets: Echinococcosis. https://www.who.int/news-room/fact-sheets/detail/echinococcosis. Accessed 15 Sep 2021.

[7] Lewall D, McCorkell S (1986). Rupture of echinococcal cysts: diagnosis, classification, and clinical implications. Am J Roentgenol.

[8] Dadoukis J, Gamvros O, Aletras H (1984). Intrabiliary rupture of the hydatid cyst of the liver. World J Surg.

[9] Atli M (2001). Intrabiliary rupture of a hepatic hydatid cyst: associated clinical factors and proper management. Arch Surg.

[10] Köksal N, Müftüoglu T, Günerhan Y, Uzun MA, Kurt R (2001). Management of intrabiliary ruptured hydatid disease of the liver. Hepatogastroenterology.

[11] Gulalp B, Koseoglu Z, Toprak N, Satar S, Sebe A, Gokel Y (2007). Ruptured hydatid cyst following minimal trauma and few signs on presentation. Neth J Med.

[12] Navsaria PH, Forlee MV, Nicol AJ (2002). Traumatic rupture of a hepatic hydatid cyst. S Afr J Surg.

[13] Gunay K, Taviloglu K, Berber E, Ertekin C (1999). Traumatic rupture of hydatid cysts: a 12-year experience from an endemic region. J Trauma.

[14] Kantarci M, Onbas O, Alper F, Celebi Y, Yigiter M, Okur A (2003). Anaphylaxis due to a rupture of hydatid cyst: imaging findings of a 10-year-old boy. Emerg Radiol.

[15] Elmali M, Ceyhan M, Ilgar M, Koprulu C, Ozfindik M, Sancak R (2009). Hepatic hydatid cyst rupture and anaphylaxis after a fall. Indian J Pediatr.

[16] Yahya AI, Przybylski J, Foud A (1997). Anaphylactic shock in a patient with ruptured hydatid liver cyst owing to trivial abdominal trauma. J R Coll Surg Edinb.

[17] Koc C, Akbulut S, Sahin TT, Tuncer A, Yilmaz S (2020). Intraperitoneal rupture of the hydatid cyst disease: single-center experience and literature review. Ulus Travma Acil Cerrahi Derg.

[18] Ayles HM, Corbett EL, Taylor I, Cowie AGA, Bligh J, Walmsley K (2002). A combined medical and surgical approach to hydatid disease: 12 years’ experience at the hospital for tropical diseases. London Ann R Coll Surg Engl.

